# Expression of Concern: microRNA-217 suppressed epithelial-to-mesenchymal transition through targeting PTPN14 in gastric cancer

**DOI:** 10.1042/BSR-20193176_EOC

**Published:** 2020-06-26

**Keywords:** microRNA-217, PTPN14, gastric cancer, EMT

The Editorial Office has been made aware of potential issues surrounding the scientific validity of this paper, hence has issued an expression of concern to notify readers whilst the Editorial Office investigates.

